# Can Aptameric Ligands Specific to Plasma Coagulation Factor VII Bind the Recombinant Form with High Affinity: Affinity Measurement by Fluorescence Method

**Published:** 2017

**Authors:** Maryam Tabarzad, Marzieh Jafari, Nastaran Nafissi-varcheh

**Affiliations:** 1. Protein Technology Research Center, Shahid Beheshti University of Medical Sciences, Tehran, Iran; 2. Department of Pharmacology and Toxicology, Faculty of Pharmacy, Ahvaz Jundishapur University of Medical Sciences, Ahvaz, Iran; 3. Department of Pharmaceutical Biotechnology, Faculty of Pharmacy, Shahid Beheshti University of Medical Sciences, Tehran, Iran

**Keywords:** Affinity, Aptamer, Factor VIIa, Fluorescence

## Abstract

**Background:**

Among diverse protein purification systems, affinity chromatography is the most attractive one in the purification process of coagulation factors. Coagulation factor VII is a plasma serine protease that has a significant role in natural human hemostasis and its recombinant form such as AryoSeven^™^, has been applied in clinical treatment of bleeding disorders. Immunoaffinity chromatography is the purification method of choice that is currently applied in the development of coagulation factor VIIa products. Aptamers as nucleic acid based affinity ligands are more promising than monoclonal antibodies. In addition, DNA aptamers are more acceptable than RNA ones in this regard.

**Methods:**

In this study, two of the aptameric DNA oligonucleotides that showed acceptable affinities for purification of coagulation factor VIIa from plasma, were selected to evaluate their affinity against Aryoseven. A serial dilution of fluorescence labeled aptamers was incubated against the concentration of 1 nM from Aryoseven. Then, a fluorescence index was calculated according to the fluorescence intensity data measured from test and control samples. The dissociation constant of aptamers was calculated according to the fluorescence index using Prism5 software.

**Results:**

Results showed that the binding affinity of the 44 nucleotide aptamer was more than 81 nucleotide aptamer sequence. As a result, this aptamer could be optimized in order to develop aptamer based affinity chromatography process for this form of recombinant coagulation factor VIIa.

**Discussion:**

Aptamers with shorter length of sequence could show higher affinity in target binding, as they could adapt more easily to suitable conformation according to target interaction. However, it should be considered that the selectivity of affinity ligands is also important for target purification and analytical applications.

## Introduction

Aptamers are small ligands with high affinity and selectivity to their targets, which have been developed by *in vitro* selection process against a wide range of target molecules ^1–3^. Aptamers are appropriate ligands for affinity separation and purification of biomolecules using affinity chromatography ^4^. Aptamers as oligosorbents have been applied in the purification of various targets ^5–9^.

Coagulation factors are the first interesting protein targets in aptamer development ^10^. One of the valuable members, involved in extrinsic pathway, is coagulation factor VII and related biopharmaceuticals have been administered as a replacement therapy in hemophilia and non-hemophilia bleeding disorders ^11–13^. Coagulation factor VII specific RNA aptamers have been selected by independent scientists to inhibit coagulation process ^14,15^.

DNA aptamers are more appropriate for analytical applications such as protein detection and purification, because of more stability than RNA aptamers. There is one patent report of DNA aptamers specific to coagulation factor VIIa developed for affinity purification of this protein from plasma samples ^16^.

Aryoseven^®^ is a recombinant form of coagulation factor VIIa and a biosimilar of Novoseven^®^, which is manufactured in Iran. Development of an efficient affinity chromatography technique for protein purification would result in a decrease in time and cost of product manufacturing ^17^. Aptamers as affinity ligands are valuable choices for affinity chromatography ^4,8,18^.

In this study, binding affinities of two aptameric sequences against Aryoseven^®^ were determined by a fluorescent method to evaluate their potentials for the development of protein purification process.

## Materials and Methods

### Chemicals and oligonucleotide sequences

Two DNA sequence of 44 and 81 nucleotides were supplied by chemical synthesis. The FAM labeled sequences of Apt81: 5′GGGAGATAGCCACGACC TATGCAGCCAGCCGCAGTGTAAGTGAATGCAG ACATGGTCTAAGTGTCCAGGCTGTGCGAAAGC 3′ and Apt 44:5′CCG CACACCACGCGCATAATC CCGCGCACACGACTTGAAGTAGC3′ were purchased from SinaClon (Iran). Recombinant coagulation factor VIIa (Aryose-ven^®^) was gifted from Aryogene company (Iran). All chemicals of molecular biology grade were supplied from Sigam-Aldrich (USA).

### Binding affinity measurement

The oligonucleotides were dissolved in binding buffer (Tris 40 *mM*, NaCl 117 *mM*, CaCl_2_ 5 *mM*, MgCl_2_ 5 *mM* and pH=7.4) to the final concentration of 100 *μM*. The solutions were thermally treated before analysis (5 *min* at 95°*C*, 10 *min* on ice, 10 *min* in room temperature). At each run, a serial dilution of aptamers was prepared from 100 *pM* to 10 *μM* in two set and duplicate tests. The protein solution was added to the final concentration of 1 *nM*. Controls were aptamer free and protein free samples. After incubation at 4*°C* for 1 *hr*, fluorescence intensities of the samples were measured at 492 *nm* excitation and 521 *nm* emission wavelength using Cytation5 Multi-Mode Reader (BioTek, USA). Fluorescent index was calculated according to the equation 1.

A *aptamer*, A *protein* and A *test* are the absorption of aptamer control, protein control and test samples, respectively, at the 521 *nm* that is the emission wavelength of fluorescence label.

$$\text{Flourescence}\;\text{Index}:\frac{\left[(\text{A}\;\text{aptamer}+\text{A}\;\text{protein})-\text{A}\;\text{test}\right]}{\text{A}\;\text{aptamer}}$$

### Statistics and dissociation constant determination

Mean±SE of duplicated tests were calculated by IBM-SPSS21. The results from three independent runs during different days were analyzed. The non-linear regression was performed using Prism5 to calculate the Kd according to the binding-saturation model and one-site total sub-deviation.

## Results

### Binding affinity characterization

Fluorescence methods for aptamer-target binding affinity determination are simpler and faster than the other ones ^5^. In this study, changes in fluorescence intensity were measured as the consequence of aptamer binding to the target protein. Raw data from fluorescence measurement showed significant variation between duplicated runs. Therefore, mean of measurement data for each sample was calculated and then applied to a formula (Equation 1) in order to derive validated data. Similar method was evaluated in aptamer related study ^19^. Kd±SE of Apt81 and Apt44 were calculated as 938±149 and 167.47±18 *nM*, respectively.

Accordingly, lower value of the calculated Kd related to the smaller aptamer and it was revealed that adaptive changes in folding structures of aptamers could happen more easily than long sequences such as truncated aptameric sequences, as widely confirmed before ^7,20–22^. Some other studies showed that truncated aptameric sequences would have similar Kd as a full length aptamer ^8,23^. It should also be considered that small length aptamers are more useful in synthesis and application ^6^.

### Prediction of aptamer secondary structure

**Figure 1.**
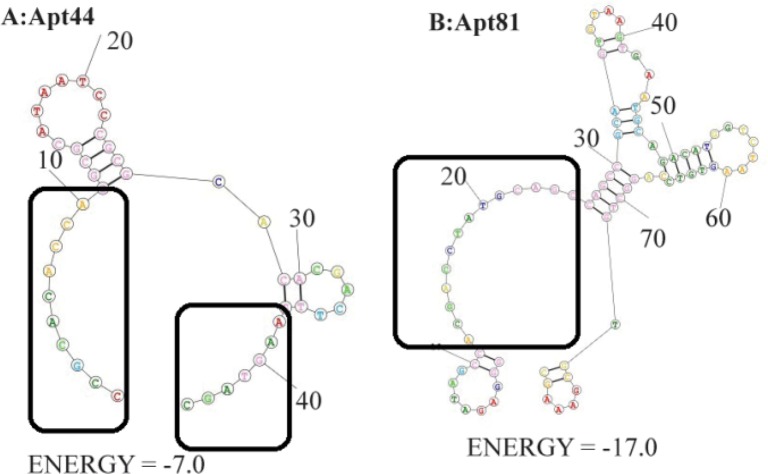
Predicted secondary structures of aptamers; A: Apt44 and B: Apt81 predicted structure by fold method. The boxes include the non-structured part

Binding affinity of aptamers is related to their 3D structure. Accordingly, secondary structure of the 44nts and 81nts aptamers was predicted by RNAStructure^®^ online server, setting the parameters for DNA entry. Predicted structures using fold method (structure with minimum free energy) were presented in figure 1. As presented, a larger part of the Apt44 nucleotide sequence was predicted as the non-structured part that could be adapted to a proper binding conformation during protein interaction. Consequently, this short aptameric ligand had more flexible structures for binding.

## Discussion

The binding affinity between nucleic acid aptamers and the target molecules could be measured by different methods. Fluorescence methods including measurement the fluorescent intensity using labeled aptamers are more feasible one. Fluorescent intensity of the labeled aptamer might change after binding to the target protein and measurement of these changes help to determine the binding affinity and kinetic. In this study, changes in fluorescence intensity were measured as the consequence of aptamer binding to the target protein, recombinant form of coagulation factor VIIa. The smaller calculated Kd of smaller aptamer revealed that adaptive changes in folding structures of aptamers happen in short sequences more easily than long sequences. Presence of non-structured positions that could be adapted to a proper binding conformation during protein binding led to flexible structures for binding, but, it would be undesirable in the development of an aptamer based affinity chromatography process if this flexibility resulted in non-specific binding to unrelated target structures. Therefore, cross binding studies should be performed to indicate that the aptamer has significant binding affinity to the background impurities or not.

## Conclusion

The study showed that plasma coagulation factor VIIa specific aptamers could bind to the recombinant form of protein with a considerable affinity, because of the structural similarities between same proteins of different sources. In addition, short length aptamers have more flexibility and higher affinity in target binding. However, during the process of designing the purification or analysis based on aptamers, it should be considered that more adaptive structure of small length aptamers could result in less selectivity of binding.
